# Experiences with a new biplanar low-dose X-ray device for imaging the facial skeleton: A feasibility study

**DOI:** 10.1371/journal.pone.0235032

**Published:** 2020-07-02

**Authors:** Britt-Isabelle Berg, Aurélien Laville, Delphine S. Courvoisier, Philippe Rouch, Thomas Schouman

**Affiliations:** 1 Department of Cranio-Maxillofacial Surgery, University Hospital Basel and University Basel, Basel, Switzerland; 2 Laboratoire de Biomécanique, Institut de Biomécanique Humaine Georges Charpak, Arts et Métiers ParisTech, Paris, France; 3 CRC & Division of Clinical Epidemiology, Faculty of Medicine, University of Geneva & University Hospitals of Geneva, Geneva, Switzerland; 4 Department of Maxillofacial Surgery, APHP—Groupe Hospitalier Pitie-Salpetriere, Universite Paris 6—UPMC, Paris, France; New York University Langone Health, UNITED STATES

## Abstract

**Background:**

This study aimed to evaluate the feasibility of a new biplanar low-dose X-ray device for facial skeletal imaging.

**Methods:**

We evaluated 48 biplanar radiographs from 12 patients (posteroanterior/lateral), originally taken for a scoliosis examination with a biplanar low-dose X-ray device. For this study, the images were further evaluated for the perceptibility of 38 facial skeleton landmarks. To determine the reliability and reproducibility of perceptibility, two independent observers determined the landmarks twice, during a time interval of at least two weeks.

**Results:**

Both interoperator and intraoperator reliability were excellent for all landmarks [intraclass correlation coefficient (ICC) > 0.92].

**Conclusions:**

The biplanar low-dose X-ray device demonstrated good feasibility for precisely assessing the anatomical landmarks of the facial skeleton. Given its excellent precision, the biplanar low-dose X-ray device data sets should be forwarded from the treating orthopedic surgeon or neurosurgeon to the orthodontist or dentist for further assessment in their field.

## Introduction

In 1992, the Nobel Prize in Physics was awarded to Georges Charpak “for his invention and development of particle detectors, in particular the multiwire proportional chamber” [1]. This technology was used to develop a new biplanar X-ray imaging system. Illés & Somoskeöy published the detailed technology behind the system, stating that the method is highly sensitive, and could potentially detect even one photon. In addition, it is less affected by scattered radiation [2]. Until now, the biplanar low-dose X-ray imaging system was used primarily in orthopedics to image the spine, hip, shoulder, joints, and extremities [3, 4]; furthermore, its role in the detection of bone lesions has been assessed [5]. Since the biplanar X-ray imaging system produces head-to-foot images, while the patient maintains a standing position, it frequently captures the facial skeleton [6–8], even when it is of no relevance for an initial patient-related assessment. Moreover, use of these images to assess dentofacial structures could enhance safety by avoiding additional exposure to radiation and reduce healthcare costs. The imaging system simultaneously acquires posteroanterior and lateral images. Both views are of great interest for dentists, orthodontists, maxillofacial surgeons, and pediatric surgeons, due to the use in cephalometric analyses. Cephalometric analyses based on landmarks allow orthodontists and maxillofacial surgeons to assess the severity and cause of dentofacial deformities, growth patterns of the facial skeleton, the ability to set a treatment plan, and eventually assess the efficacy of the treatment and its stability. Facial asymmetry can include a distortion of the entire face, if there appears to be a relationship between facial asymmetry and adolescent idiopathic scoliosis [9]. Panoramic radiographs and cephalograms, rather than standard X-rays, are routine diagnostic tools in these cases [10]. The analysis is based on a number of specific landmarks on frontal and lateral cephalograms. Thus, patients (mainly children or young adults) will be subject to repeated radiation exposure of the head over a couple of years for orthodontic or orthognathic treatment.

Keeping radiation exposure as low as possible is necessary, as radiation accumulates over an individual’s lifetime. An association between dental X-rays and the risk of intracranial meningioma has been reported by Claus et al. [11]: they found that a history of exposure from dental X-rays during an earlier period (when radiation exposure was greater than now) appears to be associated with an increased risk of intracranial meningioma. A plain dental X-ray on average has a similar radiation dosage [12] as X-rays captured for cephalometric measurements. Therefore, it is sensible to use X-rays that are already available, even if they are not captured for the purpose of cephalometric analyses. Until now, no other research group assessed the feasibility of cephalometric analysis based on this biplanar imaging or any other biplanar low-dose X-ray system that captured both views of the face at the same time for precise depiction of the facial skeleton. One publication assessed cephalograms in posteroanterior and lateral views with a dry skull, but the X-rays were not captured at the same time [13]. Therefore, we investigated whether this low-dose biplanar X-ray imaging system would be a useful tool for facial skeletal assessments. Our hypothesis was that biplanar X-ray imaging quality was acceptable for sufficient facial landmark placement, as used in orthodontic planning and orthognathic surgery. Our aim was determine if the biplanar low-dose X-ray imaging of the facial skeleton was sufficient for landmark assessment, even if artifacts occur (as in the images we assessed for patients who had their hands in front of the face). We decided to review the images of the same patients, once with hands in front of the face and once without hands in front of the face (hands on the shoulders).

## Materials and methods

### Volunteers

**Fig 1:**
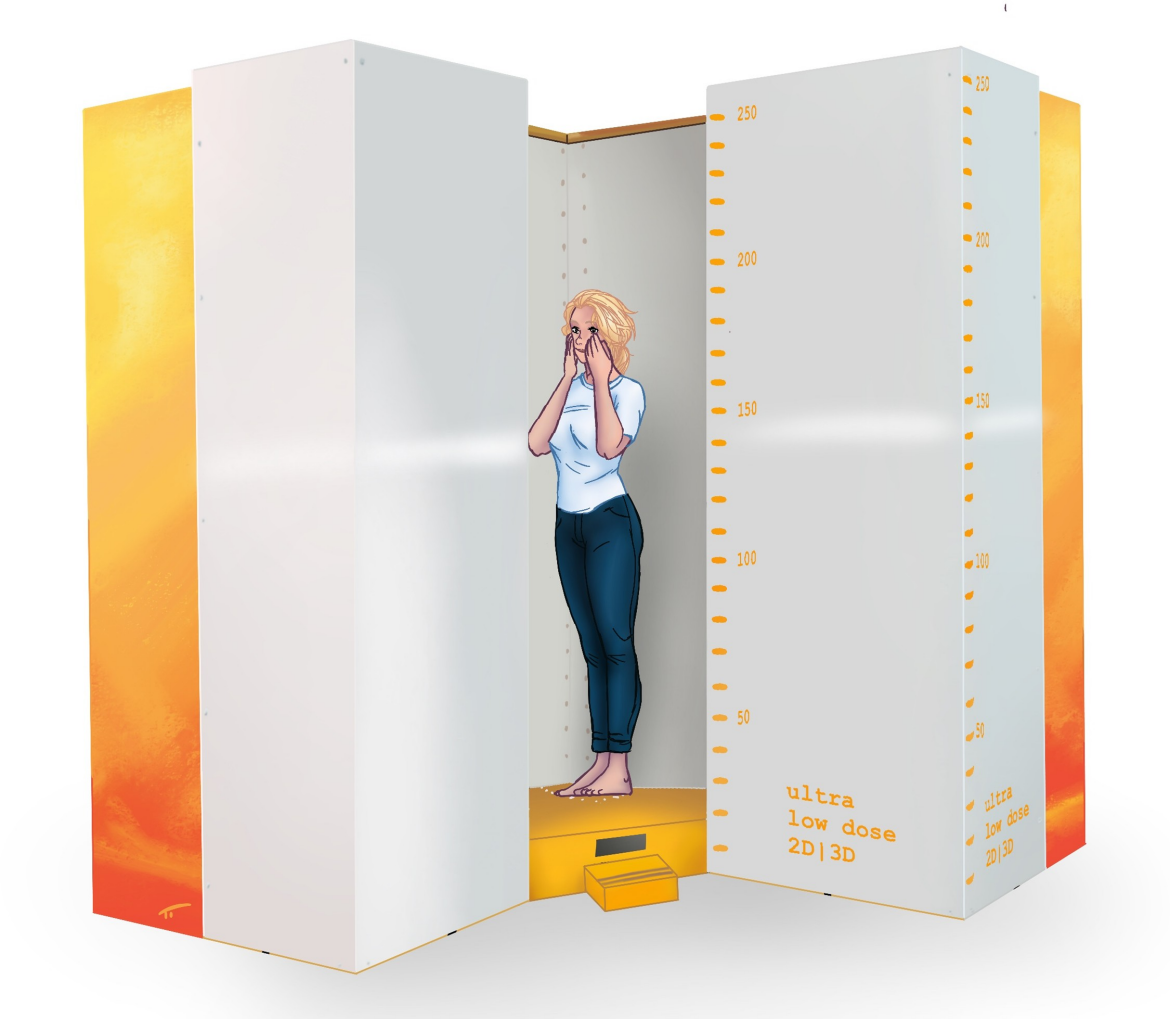
The positioning of a patient from Group A (hands in front of the face) in the biplanar low-dose X-ray device.

**Fig 2:**
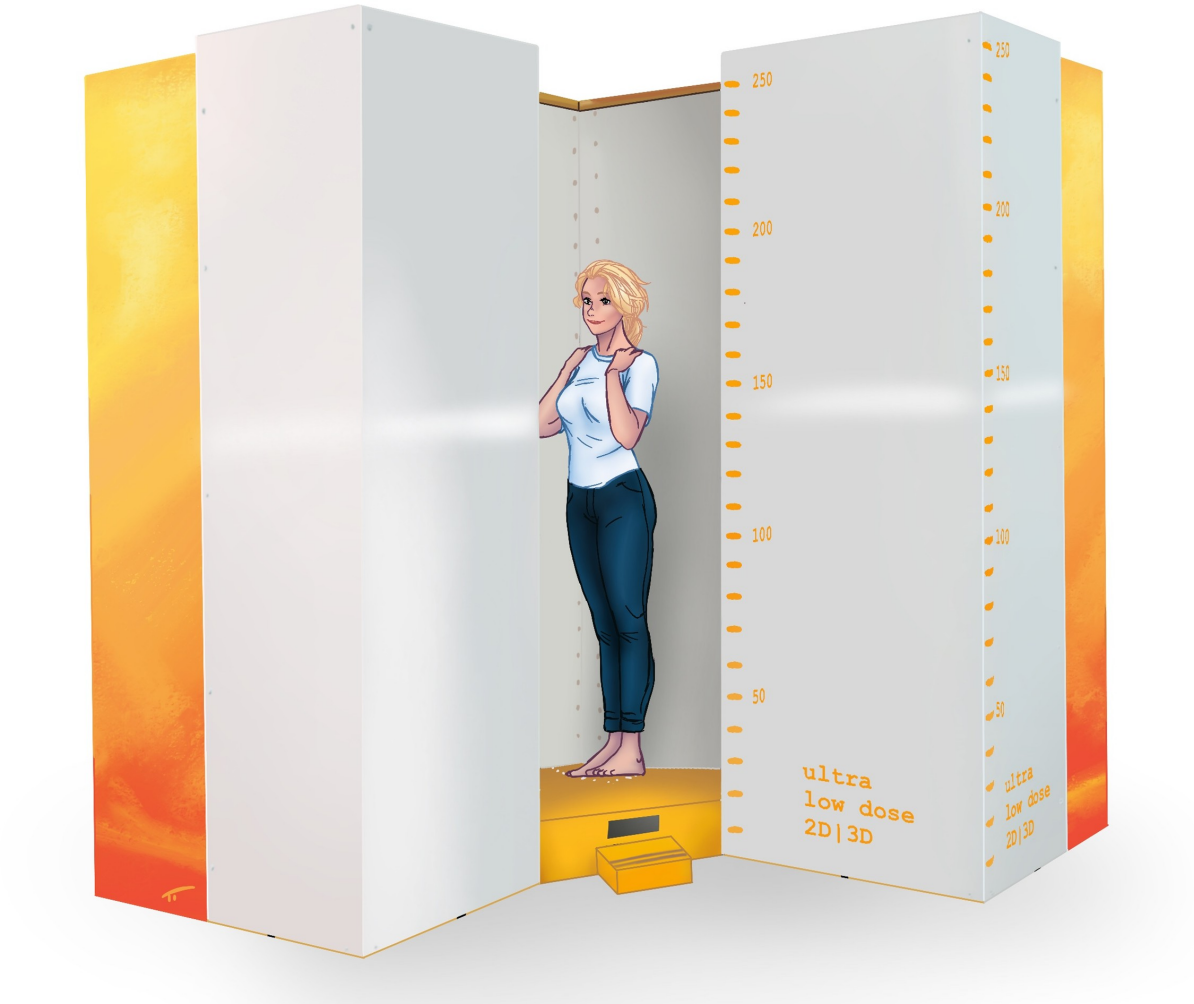
The positioning of a patient from Group B (hands on the shoulders) in the biplanar low-dose X-ray device.

Ethical approval was received from the local ethical committee "Comités de protection des personnes en Ile-de-France" (CPP IDF VI, No 90–06). Patients had provided written informed consent for the original study. We used a total of 48 biplanar radiographs from 12 patients, which were acquired with a biplanar low-dose X-ray imaging system, initially for spinal assessments with various upper extremity positions (patients provided informed consent for the original study). The images included 24 data sets (12 posteroanterior and 12 lateral) from patients in a standing position with hands in front of their face (Group A), and 24 data sets (12 posteroanterior and 12 lateral data sets) from the same patients without hands in front of their face (Group B). Depicted in Figs 1 and 2 is the exact position of the patient in the biplanar low-dose X-ray device. Fig 1 shows the patient with hands in front of the face (Group A), and Fig 2 shows the same patient with hands on the shoulders (Group B).

### The biplanar low-dose X-ray imaging system

**Fig 3:**
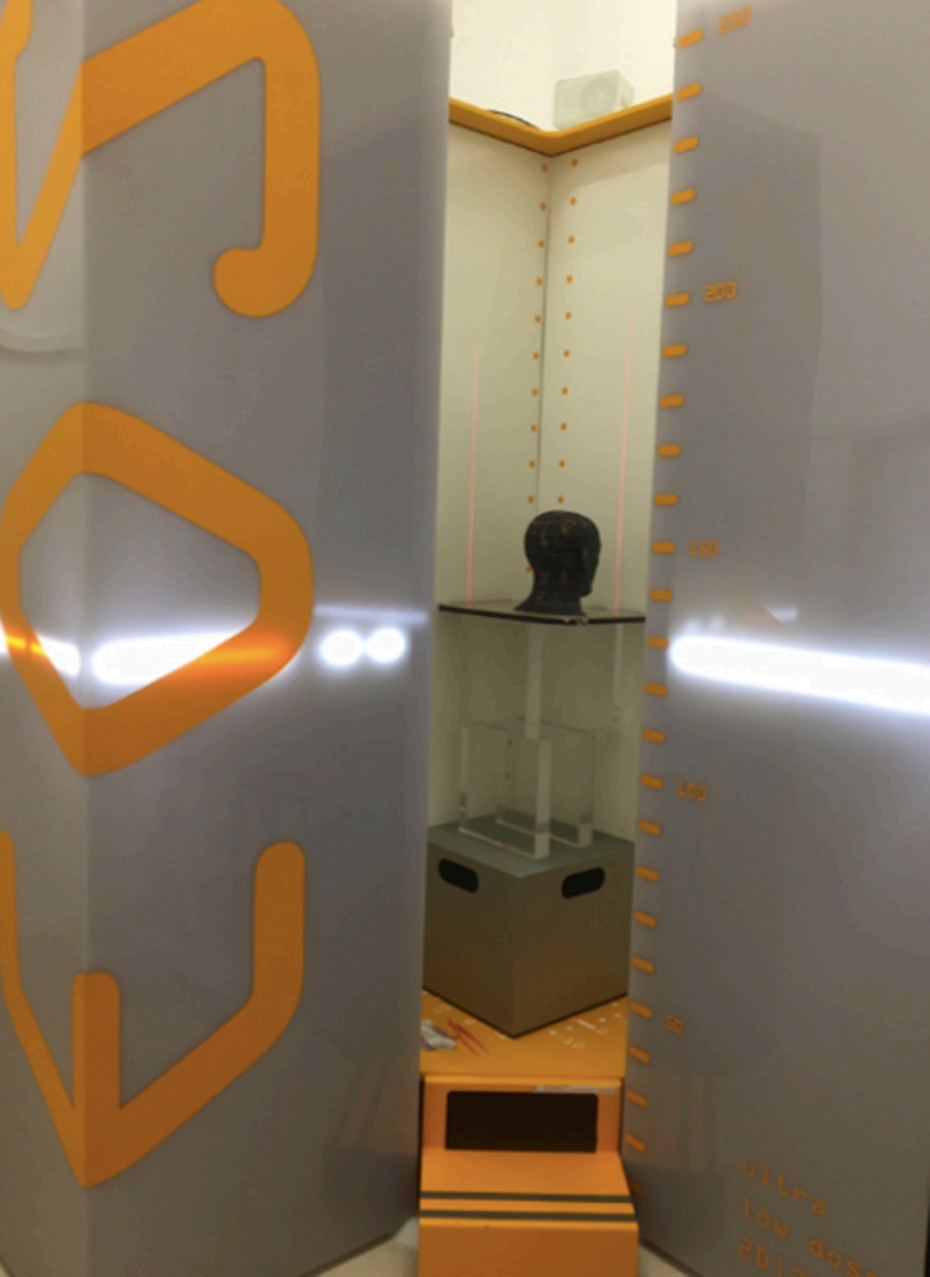
For better illustration, an Alderson head phantom was placed in the biplanar low-dose X-ray imaging system. The light outside is a laser beam, showing the height in which the image is captured at this moment. A certain distance (in cm) can be set in the system.

The device is the EOS® system (EOS®, EOS Imaging SA, Paris, France). All patients were standing. The biplanar X-ray imaging system used two perpendicular X-ray beams that were collimated in two extremely thin, horizontal, fan-shaped beams with two detectors. Since this was a retrospective study, we used X-rays of a phantom head during data acquisition to illustrate the installation (see Fig 3).

Effective resolution was reduced to 193 μm × 185 μm for the frontal view and 179 μm × 185 μm for the lateral view [14]. The reference settings were chosen as follows: Frontal: tube voltage 90 kV, current-time product 50 mAs, DAP 716 mGy cm^2^; Lateral: tube voltage 105 kV, current-time product 320 mAs, DAP 1082 mGy cm^2^. The morphology was adult, the scanned length was 76 cm, scanning speed was 5 s, and acquisition time was 12.5 s.

### Measurements

**Fig 4:**
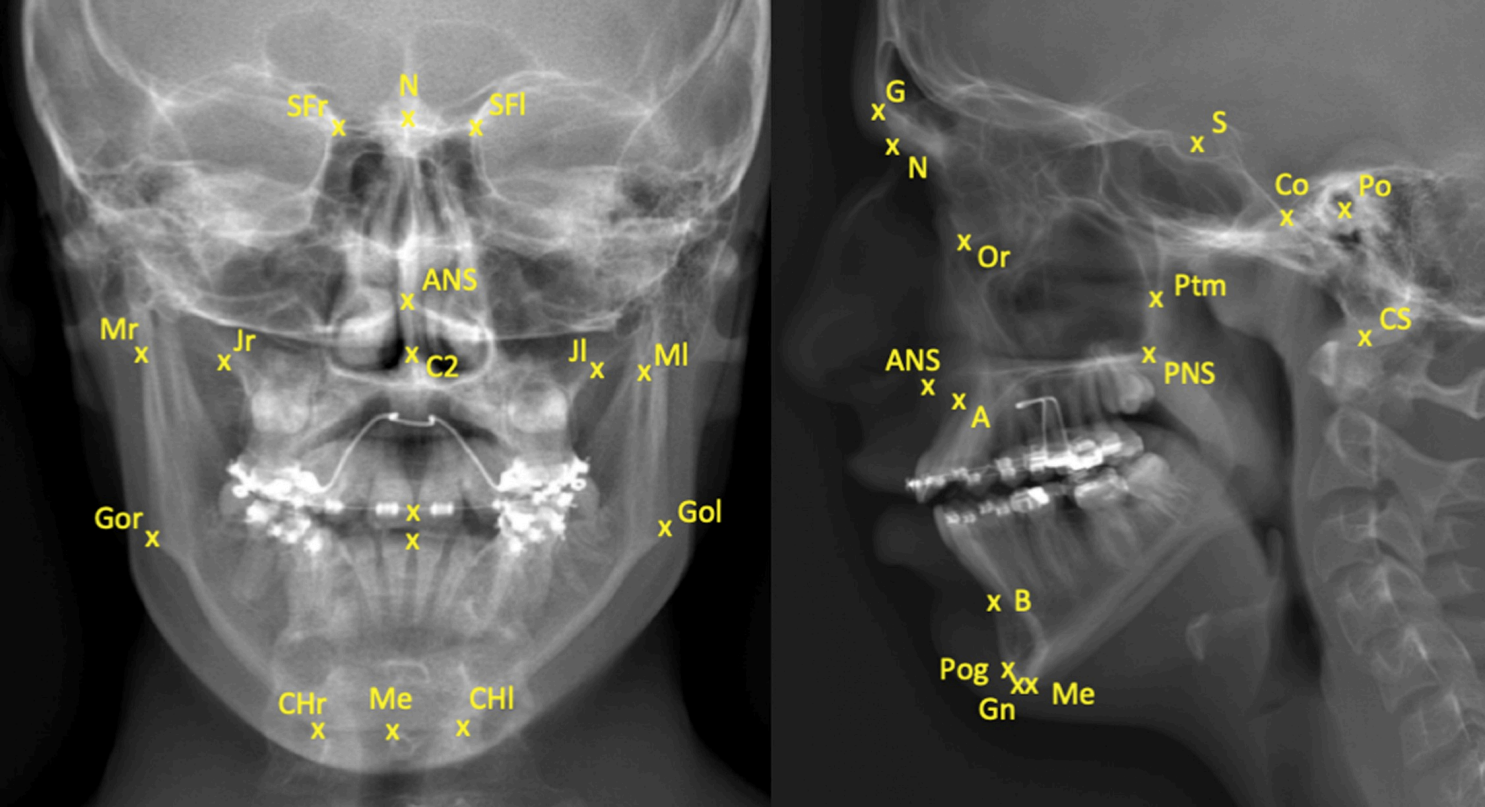
Posteroanterior and lateral images of a patient without hands in front of the face, using the biplanar low-dose X-ray imaging system. The 38 landmarks are placed to illustrate the anatomical regions.

To evaluate the feasibility of the biplanar X-ray imaging system for pictorial depiction of the facial skeleton, two craniomaxillofacial surgeons (experienced in orthognathic surgery and dentomaxillofacial radiology) placed 38 landmarks on each image. The landmarks for assessment of the frontal and lateral images were placed according to the description provided in “Essentials of Orthognathic Surgery” [15]. For both views, landmark C2 was added to represent the odontoid process. Table 1 gives detailed descriptions of the 38 anatomical regions of the landmarks as seen in Fig 4.

**Table 1:** Landmarks according to descriptions provided in “Essentials of Orthognathic Surgery” [16]. For both views, landmark “C2” was added to represent the odontoid process.

	Posterior view
**(SF)**	Where the smaller wing of the sphenoid bone crosses the medial orbital ridge
**(ANS)**	Anterior nasal spine: the center at the base of the nose
**(J)**	Jugular: the most superior and medial point of the zygomatic buttress
**(M)**	Mastoid: the most inferior point of the mastoid point
**(A)**	The contact area between the maxillary incisors
**(B)**	The contact area between the mandibular incisors
**(Go)**	Gonion: the most inferior posterior point at the angle of the mandible
**(Me)**	Menton: the most inferior posterior point at the anterior mandibular area
**(CH)**	The most inferior posterior point on the anterior inferior border of the mandible
**(C2)**	Upper border of the odontoid process
	**Lateral view**
**(G)**	Glabella: the most anterior point of the frontal bone
**(N)**	Nasion: the most anterior point of the frontal nasal suture in the midsagittal plane
**(Or)**	Orbital: the lowest point on the inferior orbital rim
**(S)**	Sella: the center of the sella turcica on the lateral cephalogram, which is located by inspection
**(Ptm)**	Pterygomaxillary: the apex of the teardrop-shaped pterygomaxillary fissure (lowest point of the opening)
**(ANS)**	Anterior nasal spine: anterior tip of the nasal spine
**(PNS)**	Posterior nasal spine: the most posterior aspect of the palate bone
**(A)-**	Point or subspinal: the most posterior midline point in the concavity where the lower anterior adage of the anterior nasal spine meets the alveolar bone, overlying the mandible incisors (infradentale) and the pogonion
**(B)-**	Point or supra-mental: the most posterior midline point in the concavity of the mandible between the alveolar bone overlying the mandible incisors (infradentale) and the pogonion
**(Pog)**	Pogonion: the most anterior point of the chin
**(Go)**	Gonion: the point defined by using two lines, with one tangential to the inferior border of the ramus; this can be assessed by bisecting the angle formed by the two lines and extending the bisectors through the curvature of the mandible
**(Gn)**	Gnathion: the lowest, most anterior midline point on the symphysis of the mandible in the midline
**(Me)**	Menton: the most inferior point on the symphysis of the mandible in the midline
**(Po)**	Porion: the most superior point of the external auditory meatus; the machine porion is the uppermost point on the outline of the rods of the cephalometer
**(Co)**	Condylion: the most posterosuperior point on the head of the condyle
**(C2)**	Upper border of the odontoid process
**(Ba)**	Basion: the median point on the anterior margin of the foramen magnum

Two independent raters were instructed to place the landmarks twice, i.e., on two separate occasions, at an interval of two weeks to assess consistency of the landmark placement at two time points, given consistency of the estimates of placement (interoperator reproducibility). The landmarks were digitally placed (computer mouse on a high-resolution monitor) with image-treatment software (Idefix, an imaging processing software based on C++, developed at the Laboratoire de Biomécanique/Institut de Biomécanique Humaine Georges Charpak, Arts et Métiers ParisTech, Paris, France) to provide coordinates of each landmark in a two-dimensional reference system. The stereo-corresponding landmarks (visible in both views: N, ANS, Orl, Orr, Ml, Mr, Gol, Gor, Me, see Table 1) were selected in one view, as the level (y-axis) of the same landmark was indicated in the other view by an epipolar line. Values in the x- and y-axis were transferred to an Excel spreadsheet for further calculation.

### Analysis

Interoperator and intraoperator reliability were evaluated for each landmark as recommended by the International Organization for Standardization [16]. One-way random effects, absolute agreement, and single rater (or single measurement) intraclass correlation coefficients [Ref. to (ICC (1,1), according to the Shrout and Fleiss convention [17]], were calculated for each landmark and presented according to group membership (Group A vs. Group B). The ICC expressed the proportion of true variance and the proportion of total variability due to differences among the participants. ICC values > 0.91, 0.71–0.91, 0.51–0.70, or < 0.51 were considered to represent excellent, good, moderate, or poor agreement, respectively [18]. The R v.3.0.1 software package (R Development Core Team, R Foundation for Statistical Computing, Vienna, Austria) was used to perform all statistical analyses. We used the moment distance to report the difference between the two observers in millimeters. Further information on this method can be found in the publication by S.D. Springate [19].

## Results

**Fig 5:**
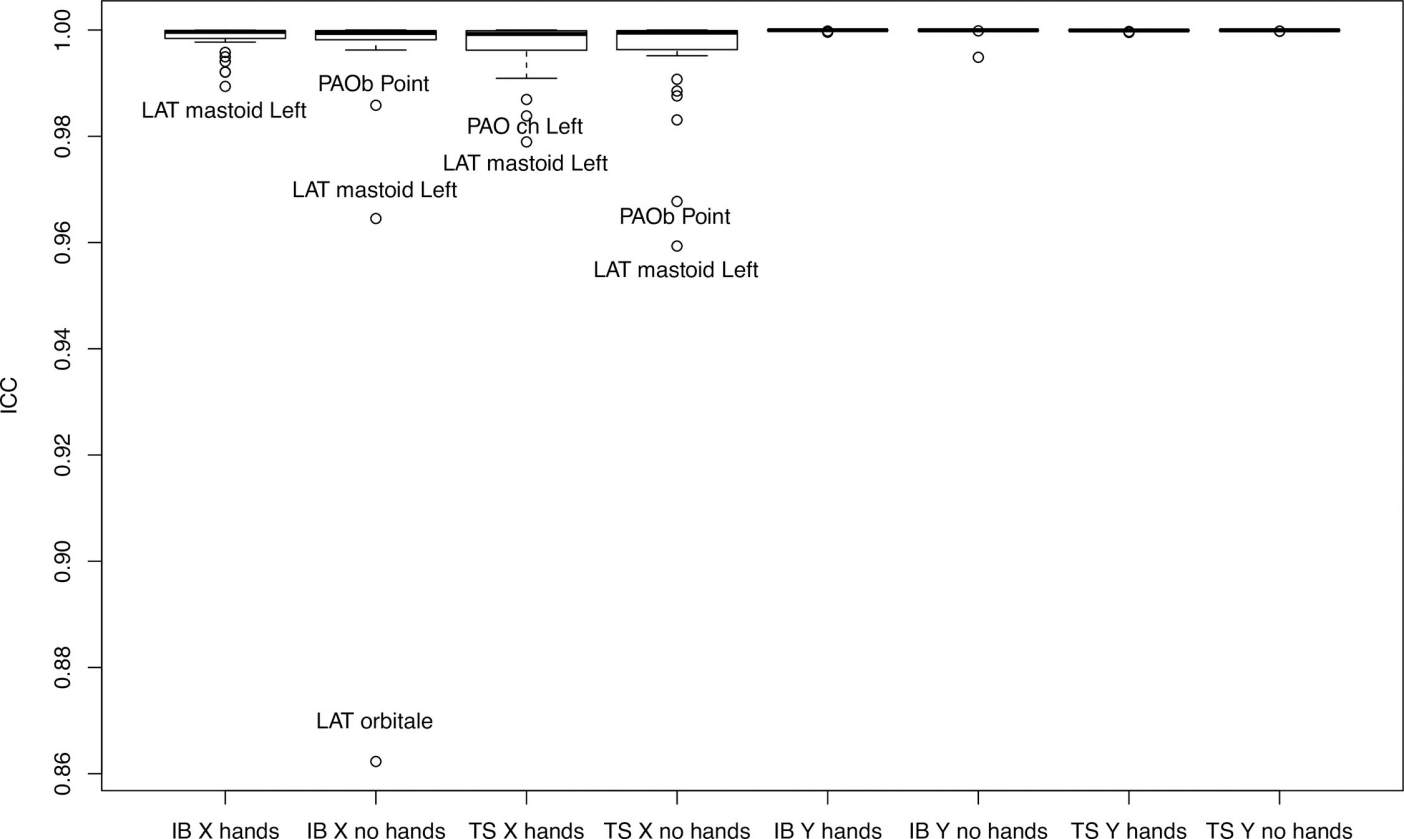
ICC values for intraoperator reliability for perceptibility of the 38 facial skeleton landmarks on the 12 biplanar radiographs of patients with hands/without hands in front of the face. IB and TS are the raters’ initials; x and y are the shortening of the x- and y-axis.

**Fig 6:**
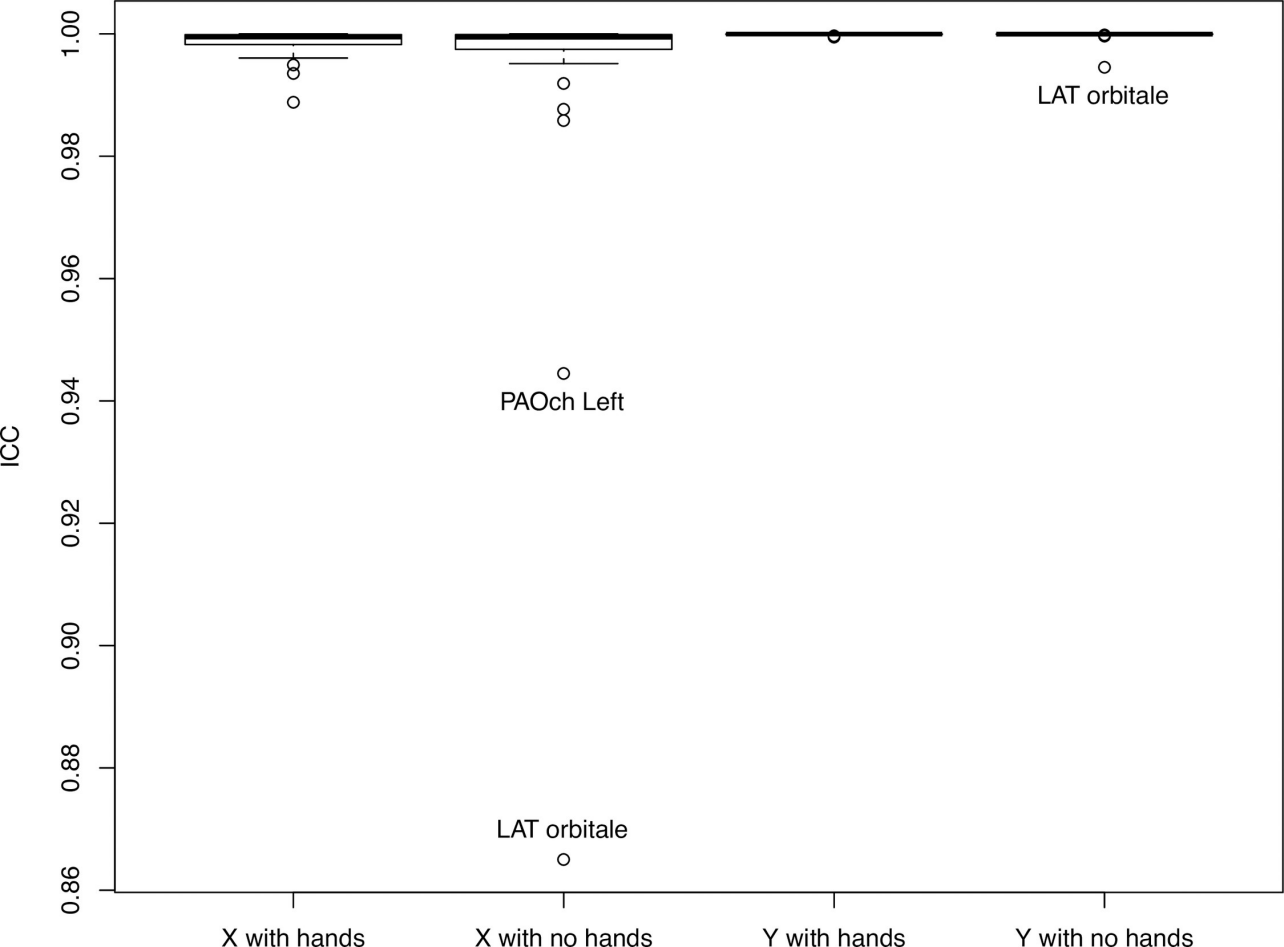
ICC values for interoperator reliability for the perceptibility of 38 facial skeleton landmarks on the 12 biplanar radiographs of patients with hands/without hands in front of the face. IB and TS are the rater’s initials; x and y are the shortening of the x- and y-axis.

The average age of the 12 volunteers was 46 (range, 17–71 years). Fig 5 (Group A, with hands) and Fig 6 (Group B, without hands) contain ICC values, averaged for x- and y-axis values, as well as for intraoperator and interoperator reliability for the perceptibility of the 38 facial skeleton landmarks on the 12 biplanar radiographs.

Both interoperator and intraoperator reliability were excellent for all landmarks (ICC > 0.92). The ICCs of Group A were not significantly different from those of Group B (interoperator: *t* = 1.39; *p* = 0.17; *d* = 0.32; intraoperator rater 1: *t* = 1.13; *p* = 0.26; *d* = 0.26; intraoperator rater 2: *t* = 0.69; *p* = 0.49; *d* = 0.17). Detailed ICC values are in S1 and S2 Tables.

The greatest difference in millimeters between the two observers, for the x and y values (in Groups A and B) was found for the Orbital (Or) landmark in the lateral view; this is likely due to the fact that one value was aberrant. Most likely, the rater did not push the button on the computer mouse strongly enough, although the overall values were still excellent. The detailed millimeter differences for each landmark are shown in S3 Table.

## Discussion

Our study was the first to investigate the feasibility of a biplanar low-dose X-ray imaging system for the facial skeleton. Image quality and excellent reproducibility/repeatability of the facial landmarks are promising. No significant differences are found between the two groups in terms of ICC values; therefore, the landmarks were precisely detectable even if patients had their hands in front of the face. This indicates the potential use of this system, not only in the field of orthopedics, but for imaging of craniofacial bony structures.

The patients in our retrospective study had been diagnosed with scoliosis. Scoliosis requires continuous follow-up with imaging. In cases of congenital scoliosis, for which surgery is indicated, annual follow-up imaging is also necessary until age 20 [20]. Additional radiation doses can be prevented with extended spinal biplanar X-ray imaging. Previously, these images were not used to assess facial asymmetries. Our assessment showed that these images were of adequate quality for evaluation of the facial skeleton, despite evidence of a correlation between facial asymmetry and scoliosis. A previous publication reported that children with scoliosis were more likely to have malocclusions [9]. Another study compared the occlusal parameters of 103 children with idiopathic scoliosis in a random group and found more asymmetric features of malocclusion relative to the control group [21]. Scoliosis patients are not the only group in which assessment of the captured faces might have demonstrated this. Proffit et al. reported that 57% to 59% of the US population is in need of some degree of orthodontic treatment [22]; this indicates that imaging for cephalometric assessment is necessary, which would expose a substantial part of the population to radiation. Given our results, biplanar X-ray imaging would be acceptable for an initial radiological assessment and follow-up when orthodontic/ maxillofacial treatment becomes necessary.

A comparison of our results with other studies using the same landmarks, but other X-ray devices, reveals that our interoperater and intraoperater reproducibility was comparable or better, even with an aberrant value; this could have occurred when the person performing the digital marking did not push the button on the computer mouse strongly enough. The rater might have thought that the mouse was correctly positioned and clicked in a certain way, but in fact, the “click” was delayed. The authors of a study on the reliability of locating landmarks of the porion (Po) and condyle (Co) compared two groups of patients, one with open mouth and one with teeth in centric occlusion. This showed that the “average reproducibility of all measurements for observers I and II was 93% and 81%” [23]. Superimposing both data sets on special foils, the set with the mouth open and the set in the centric position, were assessed in millimeters [23]. Manual setting of landmarks is still common, although landmarks are mostly digitally set. In a study conducted by Durão et al. [24], the accuracy of two-dimensional cephalometric analysis was evaluated. They used the same landmarks as in our study (though not all of them: N–Nasion; Me–Menton; ANS–Anterior Nasal Spine; Co–Condylion; Gn–Gnathion; A–Point A; B–Point B; Pog–Pogonion; Po–Porion; Or–Orbitale; Go–Gonion). They compared it to measurements of skulls, the gold standard. They found intraoperator consistency ICC above 0.9, except for one landmark in which the ICC was 0.79 [24]. Interoperator reliability with ICCs between 0.67–0.99 depend on the landmark. In their study, two observers performed the measurements. The second measurement was done after a one-month interval. Differences of < 1 mm were accepted as within one standard deviation (SD) and were considered clinically acceptable in this study [24]. Landmarks were digitally placed on the monitor [24]. Paixão et al. [25]. found no statistically significant differences (p > 0.05) when comparing cephalometric measurements of digital cephalometric tracing, with manual placement in lateral cephalometric radiographs. Comparing our results with conventional digital lateral cephalogram radiographs, reproducibility measurements in that study were slightly more precise. Comparing our results with those of Durão et al. [24], in some landmarks, our differences were > 1mm. Using the middle of the sella as an example (“S-point”), measurements published by Greiner et al. [26] yielded a mean “x”/”y” value quality range of 0.28 mm/0.21 mm. In our measurements, sella “S-point” values of 0.42 mm/ 0.42 mm were found with respect to inter-reproducibility (in Group B). An accuracy of less than 0.5 mm is precise and will be more than sufficient for the assessment used in clinical routine. The landmarks that exhibit greater differences than 1 mm are, in general, subject to interpretation in placement and are therefore more difficult to position, which might explain greater interoperator differences. The detailed verification of all landmarks of the face guarantees that the biplanar X-ray can be used for most interventions in the dentofacial area. Other studies described difficulties in identifying the “Go” and “Me” landmarks [27–29], which proved to not be the case in our study with the biplanar X-ray imaging system tool. When comparing reproducibility of landmarks/anatomical points of our results with other studies, using the biplanar low-dose X-ray imaging system, e.g., reproducibility of vertebrae angle measurements, we reported ICC values of 0.97–1.00 [18]. A study of scapular landmarks found maximum interoperator differences of 1.48–13.24 mm and average intraoperator differences of 0.25–2.53 mm [30]. Images in our study were not originally captured for detection of facial skeletal structures, but to clarify if hands in front of the face had an impact on spinal assessment. Positioning of the tongue, the Frankfurter Horizontal, a line to orient the head or the chin, were not considered. The patient was not perfectly placed in the device, yet it was possible to achieve very good image quality.

Even with hands in front of the face, these captured images could also be used by maxillofacial surgeons or orthodontists in deciding between conservative treatment and orthognathic surgery. The lateral view could be assessed to obtain the growth pattern, and the posteroanterior images could be used to assess facial asymmetries.

Since this study was retrospective, dosage measurements of the face were not performed. It should be left to future investigation whether (in regard to radiation dosage), the biplanar low-dose X-ray imaging system is superior to conventional cephalometric devices. However, the results of the present study, e.g., the high quality of landmarks assessment, promote the use of images captured with the biplanar low-dose X-ray imaging system for the same measurements, usually performed by conventional cephalometric devices, when available.

## Conclusion

The biplanar low-dose X-ray device showed good feasibility for precisely assessing the anatomical landmarks of the facial skeleton. Given such excellent precision, the biplanar low-dose X-ray device data sets should be forwarded from the treating orthopedic surgeon or neurosurgeon to the orthodontist or dentist for further assessment in their field.

## Supporting information

S1 Table ICC values for intraoperator and interoperator reliability for the perceptibility of 38 facial skeleton landmarks on the 12 biplanar radiographs of patients with their hands in front of the face. (DOCX)

S2 Table ICC values for intraoperator and interoperator reliability for the perceptibility of 38 facial skeleton landmarks on the 12 biplanar radiographs of patients without their hands in front of the face. (DOCX)

S3 Table The millimeter differences for interoperator reliability of 38 facial skeleton landmarks on the 12 biplanar radiographs of patients with their hands in front of the face (X.H.- x-value of the patient group with hands in front of the face), X.N.H.- x-value of patient group with no hands in front of the face, Y.H.- y-value of patient group with hands in front of the face, Y.N.H.- y-value of patient group with no hands in front of the face). (DOCX)
