# Supplementary material for: Experiences with a new biplanar low-dose X-ray device for imaging the facial skeleton: A feasibility study
Source: PLoS One. 2020 Jul 2;15(7):e0235032. doi: 10.1371/journal.pone.0235032 (PMC7331994; doi:10.1371/journal.pone.0235032)
Supplement: S1 Table — (DOCX) [file pone.0235032.s001.docx]

**Supporting Table #1**: ICC values for intraoperator and interoperator reliability for the perceptibility of 38 facial skeleton landmarks on the 12 biplanar radiographs of patients with their hands in front of the face.

| **Landmarks** | **ICC intraoperator rater 1** | **ICC intraoperater rater 2** | **ICC interoperator** |
| --- | --- | --- | --- |
| **Posterioanterior** |  |  |  |
| **A** | 0.99886 | 0.99540 | 0.99905 |
| **ANS** | 0.99935 | 0.99895 | 0.99905 |
| **B** | 0.99987 | 0.98945 | 0.99964 |
| **C2** | 0.99788 | 0.99748 | 0.99916 |
| **CH left** | 0.99603 | 0.99346 | 0.99441 |
| **CH right** | 0.99703 | 0.99810 | 0.99802 |
| **Go left** | 0.99995 | 0.99990 | 0.99996 |
| **Go right** | 0.99995 | 0.99993 | 0.99998 |
| **J left** | 0.99985 | 0.99967 | 0.99990 |
| **J right** | 0.99995 | 0.99972 | 0.99992 |
| **M left** | 0.99992 | 0.99953 | 0.99960 |
| **M right** | 0.99998 | 0.99991 | 0.99995 |
| **Me** | 0.99605 | 0.99545 | 0.99853 |
| **N** | 0.99744 | 0.99850 | 0.99671 |
| **Or left** | 0.99916 | 0.99732 | 0.99731 |
| **Or right** | 0.99946 | 0.99870 | 0.99904 |
| **SF left** | 0.99886 | 0.99540 | 0.99905 |
| **SF right** | 0.99969 | 0.99842 | 0.99946 |
| **Lateral** |  |  |  |
| **ANS** | 0.99996 | 0.99916 | 0.99993 |
| **A-point** | 0.99991 | 0.99978 | 0.99987 |
| **B-point** | 0.99995 | 0.99996 | 0.99989 |
| **Ba** | 0.99942 | 0.99932 | 0.99952 |
| **C2** | 0.99983 | 0.99986 | 0.99958 |
| **Co** | 0.99961 | 0.99928 | 0.99944 |
| **G** | 0.99978 | 0.99998 | 0.99993 |
| **Gn** | 0.99997 | 0.99998 | 0.99996 |
| **Go left** | 0.99937 | 0.99970 | 0.99991 |
| **Go right** | 0.99947 | 0.99970 | 0.99984 |
| **M left** | 0.99469 | 0.99191 | 0.99941 |
| **M right** | 0.99985 | 0.99785 | 0.99924 |
| **Me** | 0.99995 | 0.99993 | 0.99990 |
| **N** | 0.99989 | 0.99990 | 0.99993 |
| **Or** | 0.99906 | 0.99989 | 0.99841 |
| **Pog** | 0.99991 | 0.99996 | 0.99996 |
| **Po** | 0.99989 | 0.99855 | 0.99982 |
| **PNS** | 0.99982 | 0.99994 | 0.99980 |
| **Ptm** | 0.99956 | 0.99982 | 0.99989 |
| **S** | 0.99998 | 0.99995 | 0.99997 |
|  |  |  |  |
