# Supplementary material for: Experiences with a new biplanar low-dose X-ray device for imaging the facial skeleton: A feasibility study
Source: PLoS One. 2020 Jul 2;15(7):e0235032. doi: 10.1371/journal.pone.0235032 (PMC7331994; doi:10.1371/journal.pone.0235032)
Supplement: S2 Table — (DOCX) [file pone.0235032.s002.docx]

**Supporting Table #2**: ICC values for intraoperator and interoperator reliability for the perceptibility of 38 facial skeleton landmarks on the 12 biplanar radiographs of patients without their hands in front of the face.

| **Landmarks** | **ICC intraoperator rater 1** | **ICC intraoperater rater 2** | **ICC interoperator** |
| --- | --- | --- | --- |
| **Posterioanterior** |  |  |  |
| **A** | 0.99923 | 0.99536 | 0.99947 |
| **ANS** | 0.99969 | 0.99951 | 0.99897 |
| **B** | 0.99974 | 0.99153 | 0.99851 |
| **C2** | 0.99856 | 0.99874 | 0.99865 |
| **CH left** | 0.99291 | 0.97967 | 0.97222 |
| **CH right** | 0.99839 | 0.99817 | 0.99807 |
| **Go left** | 0.99992 | 0.99998 | 0.99995 |
| **Go right** | 0.99986 | 0.99995 | 0.99997 |
| **J left** | 0.99971 | 0.99978 | 0.99990 |
| **J right** | 0.99985 | 0.99970 | 0.99995 |
| **M left** | 0.99997 | 0.99993 | 0.99991 |
| **M right** | 0.99987 | 0.99979 | 0.99993 |
| **Me** | 0.99811 | 0.99425 | 0.99384 |
| **N** | 0.99866 | 0.99785 | 0.99752 |
| **Or left** | 0.99938 | 0.99811 | 0.99857 |
| **Or right** | 0.99963 | 0.99960 | 0.99942 |
| **SF left** | 0.99923 | 0.99536 | 0.99947 |
| **SF right** | 0.99978 | 0.99975 | 0.99989 |
| **Lateral** |  |  |  |
| **ANS** | 0.99985 | 0.99979 | 0.99986 |
| **A-point** | 0.99998 | 0.99985 | 0.99995 |
| **B-point** | 0.99996 | 0.99994 | 0.99991 |
| **Ba** | 0.99930 | 0.99876 | 0.99941 |
| **C2** | 0.99906 | 0.99949 | 0.99963 |
| **Co** | 0.99929 | 0.99953 | 0.99978 |
| **G** | 0.99992 | 0.99998 | 0.99993 |
| **Gn** | 0.99996 | 0.99998 | 0.99995 |
| **Go left** | 0.99972 | 0.99970 | 0.99950 |
| **Go right** | 0.99885 | 0.99977 | 0.99939 |
| **M left** | 0.98225 | 0.98385 | 0.99291 |
| **M right** | 0.99865 | 0.99757 | 0.99593 |
| **Me** | 0.99996 | 0.99996 | 0.99993 |
| **N** | 0.99995 | 0.99988 | 0.99995 |
| **Or** | 0.92858 | 0.99976 | 0.92979 |
| **Pog** | 0.99992 | 0.99996 | 0.99994 |
| **Po** | 0.99973 | 0.99375 | 0.99983 |
| **PNS** | 0.99931 | 0.99988 | 0.99962 |
| **Ptm** | 0.99968 | 0.99992 | 0.99982 |
| **S** | 0.99994 | 0.99995 | 0.99994 |
