# Supplementary material for: Experiences with a new biplanar low-dose X-ray device for imaging the facial skeleton: A feasibility study
Source: PLoS One. 2020 Jul 2;15(7):e0235032. doi: 10.1371/journal.pone.0235032 (PMC7331994; doi:10.1371/journal.pone.0235032)
Supplement: S3 Table — (DOCX) [file pone.0235032.s003.docx]

**Supporting Table 3**: The millimeter differences for interoperator reliability of 38 facial skeleton landmarks on the 12 biplanar radiographs of patients with their hands in front of the face (X.H.- x-value of the patient group with hands in front of the face), X.N.H.- x-value of patient group with no hands in front of the face, Y.H.- y-value of patient group with hands in front of the face, Y.N.H.- y-value of patient group with no hands in front of the face).

| **Landmarks** | **Int X.H.** | **X.N.H.** | **Y.H.** | | **Y.N.H.** | | |
| --- | --- | --- | --- | --- | --- | --- | --- |
| **Posterioanterior** |  |  |  | |  | |  |
| **A** | 0.57 | 0.41 | 0.55 | 0.39 | | |  |
| **ANS** | 0.44 | 0.49 | 0.45 | 0.90 | | |  |
| **B** | 0.25 | 0.52 | 0.75 | 0.83 | | |  |
| **C2** | 0.37 | 0.59 | 1.28 | 0.50 | | |  |
| **CH left** | 1.46 | 2.65 | 0.29 | 0.63 | | |  |
| **CH right** | 1.15 | 1.35 | 0.41 | 0.65 | | |  |
| **Go left** | 0.36 | 0.38 | 0.61 | 0.78 | | |  |
| **Go right** | 0.19 | 0.25 | 0.59 | 0.67 | | |  |
| **J left** | 0.38 | 0.37 | 0.44 | 0.52 | | |  |
| **J right** | 0.34 | 0.28 | 0.44 | 0.83 | | |  |
| **M left** | 1.44 | 0.68 | 0.54 | 0.52 | | |  |
| **M right** | 0.52 | 0.67 | 0.41 | 0.36 | | |  |
| **Me** | 0.61 | 1.33 | 0.22 | 0.63 | | |  |
| **N** | 0.72 | 0.69 | 1.42 | 1.40 | | |  |
| **Or left** | 2.23 | 1.56 | 2.12 | 2.47 | | |  |
| **Or right** | 1.59 | 1.25 | 1.71 | 1.93 | | |  |
| **SF left** | 0.57 | 0.41 | 0.55 | 0.39 | | |  |
| **SF right** | 0.65 | 0.28 | 0.49 | 0.32 | | |  |
| **Lateral** |  |  |  |  | | |  |
| **ANS** | 0.99 | 0.14 | 0.47 | 0.88 | | |  |
| **A-point** | 0.77 | 0.46 | 1.19 | 0.98 | | |  |
| **B-point** | 0.29 | 0.38 | 1.28 | 1.72 | | |  |
| **Ba** | 1.17 | 0.85 | 1.13 | 1.22 | | |  |
| **C2** | 1.03 | 0.65 | 1.09 | 0.34 | | |  |
| **Co** | 0.98 | 0.57 | 1.29 | | | 0.97 |  |
| **G** | 0.19 | 0.26 | 1.53 | | | 0.82 |  |
| **Gn** | 0.62 | 0.76 | 0.53 | | | 0.82 |  |
| **Go left** | 0.51 | 1.11 | 0.59 | | | 0.78 |  |
| **Go right** | 0.76 | 1.22 | 0.69 | | | 0.65 |  |
| **M left** | 1.34 | 3.19 | 0.35 | | | 0.52 |  |
| **M right** | 1.39 | 2.37 | 0.55 | | | 0.51 |  |
| **Me** | 1.24 | 0.98 | 0.22 | | | 0.39 |  |
| **N** | 0.90 | 0.49 | 0.90 | | | 1.07 |  |
| **Or** | 4.52 | 30.52 | 2.68 | | | 9.88 |  |
| **Pog** | 0.36 | 0.35 | 1.01 | | | 1.32 |  |
| **Po** | 0.63 | 0.42 | 0.89 | | | 1.07 |  |
| **PNS** | 1.09 | 1.47 | 0.25 | | | 0.45 |  |
| **Ptm** | 0.62 | 0.76 | 0.95 | | | 1.62 |  |
| **S** | 0.37 | 0.37 | 0.26 | | | 0.57 |  |
